# Quercetin ameliorates chicken quality by activating the PI3K/PKB/AMPK signaling pathway in broilers

**DOI:** 10.3389/fvets.2022.951512

**Published:** 2022-12-12

**Authors:** Mi Wang, Bo Wang, Shuaishuai Zhou, Jiayan Liu, Han Lu, Hao Wu, Manyi Ding, Yao Li

**Affiliations:** ^1^College of Animal Science and Technology, Northeast Agricultural University, Harbin, China; ^2^College of Animal Husbandry and Veterinary Medicine, Jinzhou Medical University, Jinzhou, China

**Keywords:** quercetin, chicken quality, PI3K, PKB, AMPK

## Abstract

This study was conducted to investigate the effects and mechanism of quercetin on chicken quality in broilers. We selected 480 AA broilers (1 day old) and randomly allotted those to four treatments (negative control and 0.2, 0.4, or 0.6 g of quercetin per kg of diet) for 42 days. Compared with the control group, the supplementation with 0.4 g of quercetin significantly increased the pH_45min_ and L^*^ value of the thigh muscle and decreased the shearing force of the thigh muscle and breast muscle and drip loss of the thigh muscle (*P* < 0.05). The supplementation with 0.6 g/kg of quercetin significantly increased the pH_45min_ and L^*^ value of the thigh muscle, and pH_45min_ of breast muscle and decreased the drip loss of the thigh muscle (*P* < 0.05). Sensory scores of meat color, tenderness, and juiciness also were improved with increasing quercetin concentration (*P* < 0.05). The inosinic acid (IMP) content of the breast and thigh muscles of broilers was significantly increased by supplementation with 0.6 g/kg of quercetin (*P* < 0.05). Supplementation with 0.2, 0.4, and 0.6 g of quercetin significantly reduced mRNA expression of L-FABP (*P* < 0.05, *P* < 0.05, and *P* < 0.05); supplementation with 0.4 and 0.6 g/kg of quercetin significantly increased mRNA expression of PKB and AMPKα1 (*P* < 0.05 and *P* < 0.05); supplementation with 0.6 g/kg of quercetin in the diet significantly reduced mRNA expression of SREBP1 and HMGR (*P* < 0.05 and *P* < 0.05) and significantly increased mRNA expression of CPT1 and PPARγ (*P* < 0.05 and *P* < 0.05); and supplementation with 0.2, 0.4, and 0.6 g/kg of quercetin significantly increased mRNA expression of PI3K, LPL, and Apo A1 and significantly reduced mRNA expression of ACC and FATP1 in the breast muscle of broilers (*P* > 0.05). PI3k, PKB, AMPK, SREBP1, and L-FABP were significantly and positively correlated with pH_45min_ (*P* < 0.05); PPARγ was significantly and positively correlated with shear force (*P* < 0.05); CPT1 was significantly and positively correlated with the L^*^ value (*P* < 0.05); and HMGR was significantly and positively correlated with drip loss (*P* < 0.05). In conclusion, quercetin improved the meat quality, protecting it against lipid oxidation and deposition by regulating the PI3K/PKB/AMPK_α1_ signaling pathway in the breast muscle of broilers.

## Introduction

**Figure 1:**
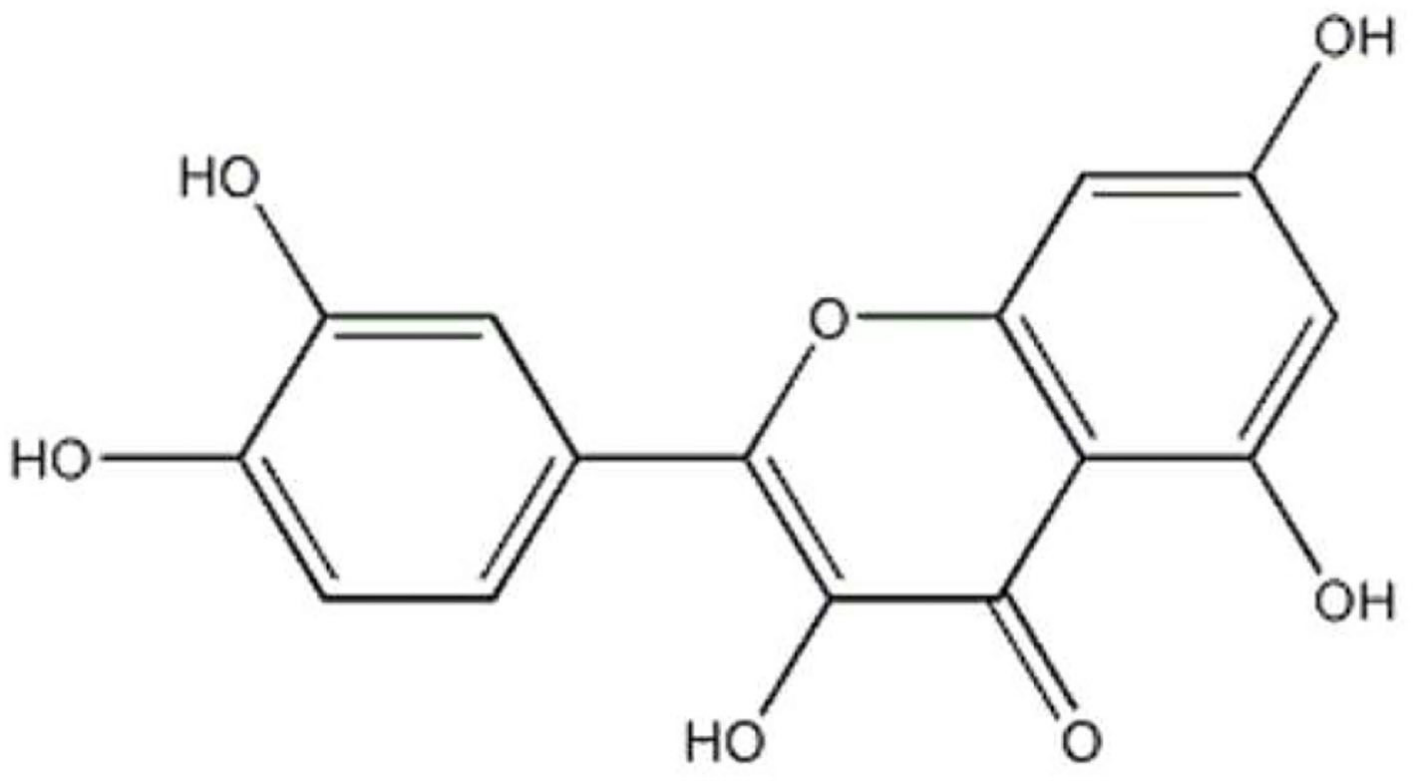
Molecular structure of quercetin.

Quercetin is a natural flavonoid found in fruits and vegetables, including red onions, tea, apples, capers, broccoli, parsley, and red grapes (1). Quercetin is present in plants, and it presents many different glycosidic forms (formed by attaching to a glycosyl group), which directly reflect the bioactivity profile of this substance. The chemical structure of quercetin is characterized by the catechol (3′, 4′o-dihydroxy) group in ring B, and by the double bond in the ring C between C-2 and C-3 in conjunction with the 4-carbonyl group as well as 3-, 5-, and 7-hydroxyl groups (Figure 1). Quercetin has been revealed to mediate a multitude of physiological functions with a broad spectrum of pharmacological properties, including anti-inflammatory, anti-diabetic, lipid modulatory, and anti-oxidative capacities (2).

5′-Monophosphate-activated protein kinase (AMPK) preserves the energy homeostasis of cells, including lipid metabolism, which is regarded as a central regulator of energy homeostasis (3). The AMPK signaling pathway might be involved in the energy metabolism alterations in the skeletal muscles of broiler chickens (4). AMPK is also a principal upstream regulator of fatty acid oxidation and lipolysis (5). AMPK may regulate fatty acid metabolism by the acetyl-CoA carboxylase (ACC)/malonyl CoA-carnitine palmitoyltransferase (CPT) pathway in the muscle of broiler chickens (6).

Hence, we hypothesized that quercetin inhibited lipid oxidation and deposition in broilers. The lipid-lowering activity of quercetin was evaluated by analyzing meat quality, chemical composition of meat, and tasting test scores. The possible mechanisms of quercetin on the suppression of lipid oxidation and deposition were investigated based on the phosphatidylinositol 3-kinase (PI3K)/serine–threonine protein kinase (PKB/AKT)/AMPK signaling pathway. To the best of our knowledge, this is the first study investigating the lipid-regulatory activity and underlying mechanisms of quercetin in meat.

## Materials and methods

### Birds, diets, and experimental design

All study procedures were performed in accordance with the guidelines set forth by the Animal Welfare Committee of Northeast Agricultural University (Harbin, People's Republic of China). Housing, management, and care of the birds conformed to the guidelines of Agricultural Animal in Agricultural Research and Teaching of Heilongjiang Province (HEI Animal Management Certificate No. 11928).

A total of 480 AA broilers (1 day old) were obtained from a commercial facility (Yi-nong Poultry, Harbin, People's Republic of China). The birds were randomly allotted to four experimental treatments, comprising six replicates of 20 birds in each treatment. All birds were raised in stainless steel cages (316 × 400 × 400 mm) under continuous light in a controlled room for 42 days. The room temperature was maintained at 33°C for the first 3 days. Then, the temperature was reduced to 24°C until the end of the experiment.

The experimental diets were based on corn and soybean meal, and quercetin was added at four concentrations: 0, 0.2, 0.4, and 0.6 g/kg of diet. Feeding was divided into two phases: the starter phase from 1 to 21 days and the grower phase from 21 to 42 days. The basal diet was formulated to meet the nutritional requirements suggested by Chinese Broiler Feeding Standards (NY/T33-2004) (Table 1). Quercetin (purity of quercetin dihydrate powder ≥97%, Sigma-Aldrich, St. Louis, MO) was mixed with the basal diet.

**Table 1:** Composition and nutrient levels of the basal diets (air-dry basis, %).

**Ingredient**	**Content** **(1–3 weeks)**	**Content** **(4–6 weeks)**
Corn	57.50	62.30
Soybean meal	34.50	30.00
Vegetable oil	3.00	3.00
Fish meal	1.00	1.00
Methionine	0.20	0.20
Dicalcium phosphate	1.62	1.67
Limestone	1.55	1.20
Sodium chloride	0.30	0.30
Multivitamin premix^1^	0.03	0.03
Mineral premix^1^	0.20	0.20
Choline	0.10	0.10
Total	100.00	100.00
**Nutrient** ^2^		
Metabolizable energy (ME), MJ/kg	12.33	12.50
CP	21.75	19.72
Total lysine (%)	1.18	1.04
Methionine (%)	0.91	0.86
Ca	1.07	0.96
Total *P*	0.70	0.68
Available *P*	0.46	0.45

1 Provided per kilogram of diet: 1,500 IU retinyl acetate, 3,200 IU cholecalciferol; 10 IU DL-tocopheryl acetate; 0.5 mg menadione sodium bisulfite; 1.8 mg thiamin mononitrate; 3.6 mg riboflavin; 3.5 mg pyridoxine hydrochloride; 0.01 mg cyanocobalamin; 0.15 mg biotin; 0.55 mg folic acid; 30 mg nicotinic acid; 10 mg pantothenic acid; 8 mg copper; 0.35 mg iodine; 80 mg iron; 60 mg manganese; 0.15 mg selenium; 40 mg zinc.

2 Based on the composition of ingredients provided by NY/T33-2004.

### Methods

#### Carcass characteristics

At the age of 42 days, 12 chickens per treatment (six per replicate pen) were randomly selected and slaughtered for carcass analyses. Each of these birds was deprived of feed for 12 h and individually weighed prior to slaughter. The percentage of the carcass, eviscerated, semi-eviscerated, breast muscle, thigh muscle, and abdominal fat was calculated according to the weight of the carcass, eviscerated, semi-eviscerated, breast muscle, thigh muscle, and abdominal fat.

#### Chicken quality

##### pH

The pH_24h_ and pH_45min_ were measured by inserting a pH meter electrode (Sentron 1001 pH System, Roden, the Netherlands) into the right breast and thigh muscle for 45 min and 24 h after slaughter, which was calibrated at pH 4.0 and pH 7.0 (7).

##### Color

The color of the right breast and thigh muscles (three measurements per sample) that were exposed to air at room temperature for 30 min was measured using a MiniScan XE (HunterLab, Reston, VA) chromameter based on the L^*^ (lightness), a^*^ (redness), and b^*^ (yellowness) values (8).

##### Warner–Bratzler shear force

Breast and thigh muscle samples of 2.54 cm thickness of from each chicken were stored at 4°C for 24 h until cooking. Steaks were placed in polyethylene bags and cooked until 71°C in the water bath, and the samples were removed and cooled in an ice slurry (1.0 ± 0.5°C) for 15 min (8). Then, six 1.27-cm-diameter cores were removed parallel with the muscle fiber orientation, and each core was sheared using a Warner–Bratzler shear force device attached to a texture analyzer (model TA-XT2i, Stable Micro Systems, UK) fitted with a tension/compression load cell of 30 kg and a crosshead speed of 240 mm/min (9).

##### Drip loss

At 24 h postmortems, the breast and thigh muscles were weighed and immediately placed in a plastic bag, hung from a hook, and stored at 2 °C for 4 days. After hanging, the samples were wiped with an absorbent paper and weighed again. The difference in weight corresponded to the drip loss and was expressed as the percentage of initial muscle weight (7).

##### Chemical composition

Proximate chemical analysis of the carcass subsections was performed. Each subsection was dried and ground separately to measure the content of crude protein and ether extract (fat).

##### Inosinic acid (IMP)

IMP concentrations in the breast and thigh muscles were determined by HPLC (4.6 × 250 mm, 5 μm particle size, Waters Corp.) (10).

##### Sensory evaluation

In total, 20 potential assessors between 18 and 33 years old (50% men and 50%women) were recruited from our department who have experience in meat quality analysis of at least 1 year. The evaluation of meat sensory attributes was performed in the breast and thigh muscles of six birds from each treatment group 2 days after the broilers were slaughtered. The breast and thigh muscle samples were cooked and randomly assigned to the 20 assessors. Color, odor, flavor, tenderness, and juiciness were tested. Score standards of the tasting trial: 1–3 is poor, 4–6 is good, and 7–9 is excellent (11, 12).

#### PI3K/PKB/AMPK signaling pathway

##### Real-time quantitative PCR analysis (RT-qPCR)

The total RNA was isolated using the TRIzol reagent. The SuperScript First-Strand Synthesis System (Life Technologies, Grand Island, NY) was adopted to synthesize first-strand cDNA from the total RNA isolated. The quantity of purified cDNAs was determined by RT-qPCR (Life Technologies, Grand Island, NY). mRNA expression levels were normalized to β-actin as reference genes. Calculations were performed using the following formulas: ΔCt (corrected sample) = mean value of target gene–mean value of internal reference gene, and ΔΔ Ct = ΔCt–mean value of the control group (Table 2).

**Table 2:** Parameters of primer pairs for the genes.

**Gene**	**Primer sequence (5^′^-3^′^)**	**Product size**	**GenBank accession**
PPARγ	F: CACTGCAGGAACAGAACAAAGAA R: TCCACAGAGCGAAACTGACATC	67bp	NM 001001460
FABP1	F: TCACTGGAAAGTACGAGC R: GCATGCAGGGTCTCTAGATT	381bp	AY 563636
FATP1	F: CGGTGCTGTTACGAGTGA R: CACGGCGTTGGAATACTT	236bp	NM 001039602
Apo A1	F: CCTTCTGGCAGCACGATGAGC	171bp	XM_015297971.2
	R: CAGCGTGTCCAGGTTGTCAGC		
LPL	F:GGTTCCTGGACAGATGGACA R:CAACATCCTTTCCCACCAGC	490bp	XM_013093728.1
PKB AMPKα1	F: CTGATGATGCCAAGGAGATT R: TGGTCAGGAGGAGTGATTGT F: AAGGTTGGCAAGCATGAGTT R: TTCTGGGCCTGCATATAACC	175bp 492bp	NM 205055.1 NM 001039603
PI3K ACC CPT1 HMGCR SREBP1 LKB β-actin	F: CGGATGTTGCCTTACGGTTGT R: GTTCTTGTCCTTGAGCCACTGAT F: CACTTCGAGGCGAAAAACTC R: GGAGCAAATCCATGACCACT F: CAATGAGGTACTCCCTGAAA R: CATTATTGGTCCACGCCCTC- F: AGCTGCAACCCTGAGGAAACT R: AGCCATCACTGTAGCACACAC F: GAGGAAGGCCATCGAGTCA R: GGAAGACAAAGGCACAGAGG F: GGGGAGACAGAAGGGAACAGA R:TGAGAGGGATGCTTGAATACGA F: TGCGTGACATCAAGGAGAAG R: TGCCAGGGTACATTGTGGTA	162bp 447bp 337bp 1268bp 392bp 158bp 300bp	NM 001004410.1 NM 205505 NM oo102898 AB109635 AY 029224 NM 001045833 L08165
18sRNA	F:TAGATAACCTCGAGCCGATCGCA	312bp	AF 173612
	R:GACTTGCCCTCCAATGGATCC TC		

### Statistical analysis

All data were subjected to one-way ANOVA with a completely randomized design with four treatments and six replicates in each treatment. The data were subjected to ANOVA, using SPSS 20.0 software. The experimental data were presented as means ± SEM, and a *P*-value of <0.05 was considered statistically significant.

## Results

### Chicken quality

Compared with the control group, the shearing force significantly reduced in the breast muscle of broilers supplemented with 0.2 g/kg of quercetin (*P* < 0.05). The shearing force of the breast and thigh muscles of broilers supplemented with 0.4 g/kg of quercetin was significantly lower than that of the control group (*P* < 0.05). The breast muscles of broilers in the 0.6 g/kg quercetin group showed significantly increased pH_45min_ (*P* < 0.01). The pH_45min_ (*P* < 0.05), L^*^ value (*P* < 0.05), and drip loss (*P* < 0.05) in thigh muscles of broilers in 0.4 and 0.6 g/kg quercetin groups were found to be significantly changed compared with those of the control group (Table 3).

**Table 3:** Effects of dietary quercetin on meat quality in AA broilers.

	**Diet (quercetin, g/kg)**			
**Items**	**0**	**0.2**	**0.4**	**0.6**
**Breast**				
pH_45min_	7.14 ± 0.51^a^	7.41 ± 0.53^a^	7.69 ± 0.16^ab^	7.83 ± 0.21^a^
pH_24h_	7.94 ± 0.25	7.96 ± 0.26	8.02 ± 0.25	7.99 ± 0.19
L*	100.03 ± 3.75	97.15 ± 5.08	93.64 ± 15.09	99.48 ± 10.95
a*	38.00 ± 3.61	41.08 ± 5.40	35.75 ± 9.14	37.26 ± 6.07
b*	51.19 ± 8.34	51.79 ± 8.36	44.58 ± 13.05	49.67 ± 7.62
Shear force (kgf)	3.34 ± 0.87^a^	2.53 ± 0.60^b^	2.34 ± 0.62^b^	3.11 ± 0.93^a^
Drip loss (%)	5.36 ± 0.40	5.68 ± 0.89	6.27 ± 0.61	6.54 ± 0.43
**Thigh**				
pH_45min_	7.23 ± 0.51^a^	7.28 ± 0.52^a^	7.63 ± 0.40^b^	7.88 ± 0.31^b^
pH_24h_	8.20 ± 0.31	8.34 ± 0.28	8.35 ± 0.32	8.48 ± 0.22
L*	68.41 ± 10.10^a^	79.10 ± 12.81^ab^	83.95 ± 11.84^b^	82.36 ± 16.69^b^
a*	57.71 ± 7.48	59.58 ± 10.48	48.60 ± 15.97	52.17 ± 8.78
b*	40.88 ± 6.12	40.01 ± 8.66	41.73 ± 16.36	41.69 ± 10.01
Shear force (kgf)	12.82 ± 3.86^a^	10.01 ± 5.63^ab^	7.06 ± 2.90^b^	10.03 ± 6.17^a^
Drip loss (%)	3.74 ± 0.22^a^	3.84 ± 0.75^a^	2.49 ± 0.27^b^	2.24 ± 0.33^b^

In the same row, values with different letter superscripts mean a significant difference (*P* < 0.05); values with no letter or the same letter superscripts mean no significant difference (*P* > 0.05). Values are expressed as means ± SEM, and n=6 for all groups. The comments are the same as below.

Compared with the control group, the protein content significantly increased in the breast and thigh muscles (*P* < 0.05), and the fat content significantly reduced in the breast muscle of broilers given 0.6 g/kg of quercetin (*P* < 0.05); the IMP content was significantly enhanced in the breast and thigh muscles of broilers in 0.4 and 0.6 g/kg quercetin groups (*P* < 0.05) (Table 4).

**Table 4:** Effects of dietary quercetin on the chemical composition of meat in AA broilers.

	**Diet (quercetin, g/kg)**			
**Items**	**0**	**0.2**	**0.4**	**0.6**
Protein of breast muscle	585.5 ± 24.2^a^	564.4 ± 17.9^a^	565.4 ± 17.6^a^	646.1 ± 13.8^b^
Protein of thigh muscle	435.8 ± 17.4^a^	451.6 ± 23.1^a^	467.6 ± 16.2^ab^	517.5 ± 28.02^b^
Fat of breast muscle (%)	6.77 ± 0.68^a^	5.45 ± 0.51^ab^	5.14 ± 0.37^ab^	4.72 ± 0.35^b^
Fat of thigh muscle (%)	25.7 ± 2.1	23.7 ± 2.35	22.6 ± 1.86	21.0 ± 1.38
IMP of breast muscle (mg/g) IMP of thigh muscle (mg/g)	2.64 ± 0.07^a^ 1.89 ± 0.08^a^	2.71 ± 0.09^a^ 2.03 ± 0.06^a^	3.16 ± 0.08^b^ 2.46 ± 0.14^b^	3.48 ± 0.07^c^ 2.72 ± 0.1^b^

Comparing with the control group, the score of juiciness in the 0.4 g/kg quercetin group was significantly increased (*P* < 0.05), and scores of meat color, tenderness, and juiciness were significantly increased in the 0.6 g/kg quercetin group (*P* < 0.05) (Table 5).

**Table 5:** Effects of dietary quercetin supplementation on the tasting test score in AA broilers.

**Items**	**0**	**0.2**	**0.4**	**0.6**
Color	3.83 ± 0.39^a^	4.50 ± 0.34^a^	4.67 ± 0.40^a^	5.42 ± 0.29^b^
Odor	4.42 ± 0.29	4.42 ± 0.26	4.33 ± 0.33	4.75 ± 0.46
Flavor	4.33 ± 0.43	4.33 ± 0.26	4.33 ± 0.33	4.92 ± 0.51
Tenderness	4.25 ± 0.39^a^	4.75 ± 0.35^ab^	4.83 ± 0.52^ab^	5.58 ± 0.19^b^
Juiciness	4.50 ± 0.29^a^	4.42 ± 0.43^a^	5.75 ± 0.25^b^	6.00 ± 0.28^b^

Tasting test score standard: 1–3 is poor, 4–6 is good, and 7–9 is excellent.

### The mechanism of quercetin ameliorating carcass characteristics and chicken quality in broilers

**Figure 2:**
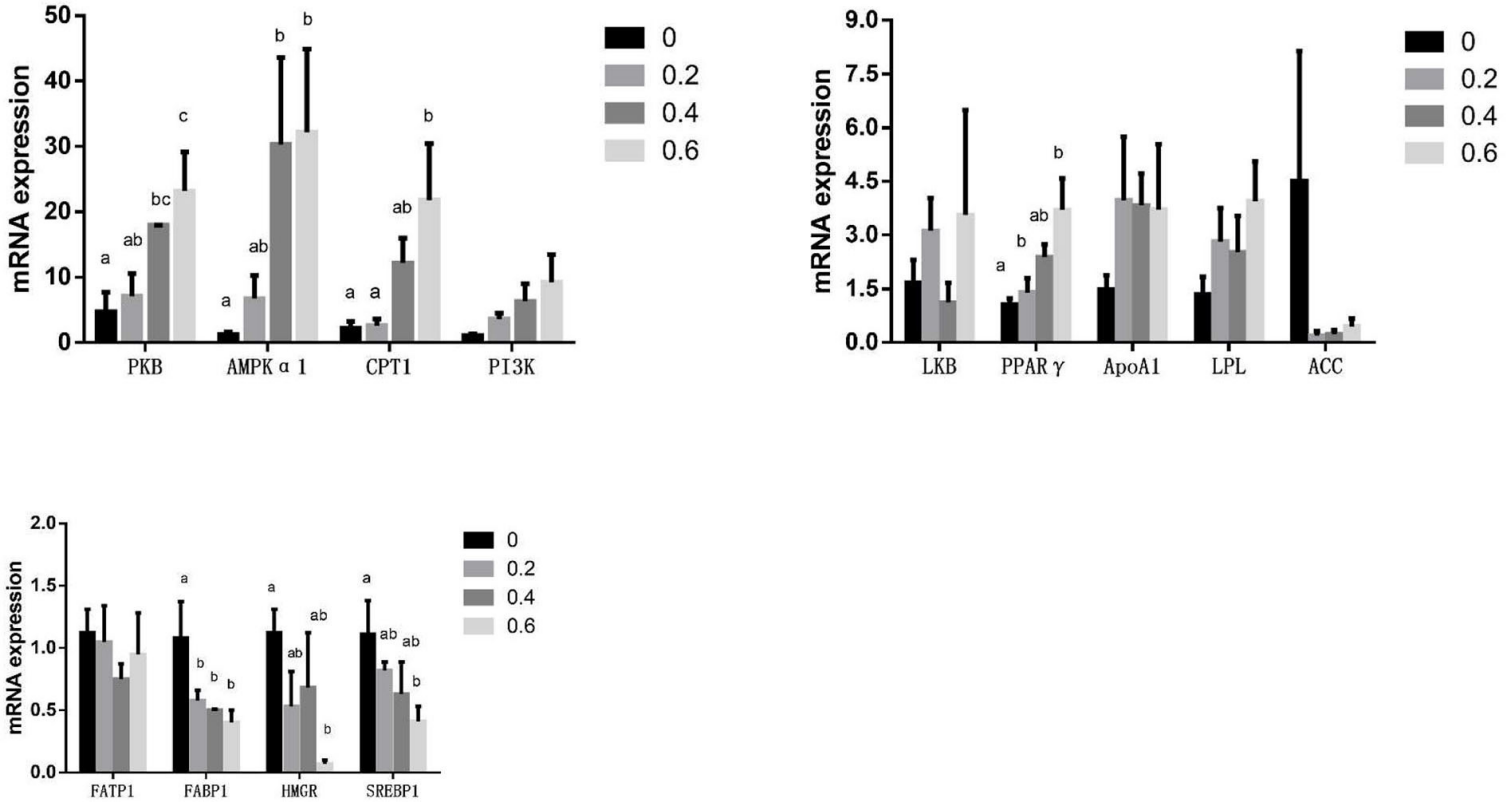
Effects of quercetin on mRNA expression of genes related to the PI3K/PKB/AMPK signaling pathway in the breast muscle of AA broilers.

The mRNA expression of FABP1(L-FABP) significantly decreased in the 0.2 g/kg quercetin group compared with the control group (*P* < 0.05). The mRNA expression of FABP1(L-FABP) was significantly downregulated, and that of PKB and AMPKα1 was significantly upregulated in the 0.4 g/kg quercetin group compared with the control group (*P* < 0.05). The mRNA expression of FABP1(L-FABP), SREBP1, and HMGR was significantly downregulated, and that of PKB, AMPKα1, CPT1, and PPARγ was significantly upregulated in the 0.6 g/kg quercetin group compared with the control group (*P* < 0.05). The mRNA expression of PI3K, LPL, and Apo A1 was upregulated, and that of ACC and FATP1 was downregulated in the breast muscle of broilers in 0.2, 0.4, and 0.6 g/kg quercetin groups (*P* > 0.05) (Figure 2).

### Correlation between chicken quality and PI3K/PKB/AMPK signaling pathway

The correlation coefficient is great than 0.6, suggesting that a correlation exists between the chicken quality and PI3K/PKB/AMPK signaling pathway—the larger the value, the stronger the correlation. PI3k, PKB, AMPK, SREBP1, and L-FABP were significantly and positively correlated with pH_45min_ (*P* < 0.05). PPARγ was significantly and positively correlated with shear force (*P* < 0.05). CPT1 was significantly and positively correlated with the L^*^ value (*P* < 0.05). HMGR was significantly and positively correlated with drip loss (*P* < 0.05) (Table 6).

**Table 6:** Correlation between chicken quality and PI3K/PKB/AMPK signaling pathway.

	**pH_45min_**	**Drip loss**	**Shear force**	L*
PI3K	0.487*	0.296	0.197	0.391
PKB	0.512*	0.139	0.419	0.114
AMPKα1	0.569*	0.246	0.045	0.102
LKB1	0.064	0.073	0.255	0.147
CPT1	0.073	0.135	0.108	0.492*
SREBP1	0.502*	0.141	0.145	0.332
ACC	0.209	0.201	0.207	0.191
PPARγ	0.188	0.136	0.477*	0.002
LPL	0.105	0.304	0.211	0.329
FATP1	0.337	0.07	0.403	0.28
FABP1	0.509*	0.005	0.172	0.338
Apo A1	0.367	0.022	0.001	0.193
HMGR	0.149	0.726**	0.113	0.06

Data are presented as mean ± standard deviation (*n* = 6). Significant differences (*P* < 0.05) are indicated by asterisks.

## Discussion

### Chicken quality

Muscle pH is an important factor affecting meat quality. pH is a potential indicator of quality characteristics of meat; a rapid postmortem decline in pH may denature protein, resulting in pale color and low water-holding capacity. Normally, muscle pH ranges from 7.35 to 7.45 and remains stable. After slaughtering and bleeding, the physiological regulation function of the body deteriorates, and lactic acid is formed by glycolysis, which rapidly decreases muscle pH (13). The rapid decrease in muscle pH inhibits the tenderization function of the Ca^2+^-activating enzyme system, which accelerates the formation of pale, soft, and exudation (PSE) meat (14). Therefore, an increase in pH of poultry meat after slaughter may delay the formation of PSE meat to some extent. Genistein and hesperidin supplemented with 20 mg/kg significantly increased muscle pH_45min_ of broilers (15). This study showed that supplementation with 0.4 and 0.6 g/kg of quercetin could markedly significantly increase pH_45min_ in the breast and thigh muscles of broilers (*P* < 0.05). Results from this study are supported by the study conducted by Jiang (16), who reported that supplementation of broiler diets with 0.4 g/kg of quercetin increased muscle pH_45min_. Hence, quercetin delayed the formation of PSE meat, and the result is confirmed by the study by Swatland (17).

The previous studies have measured the L^*^ value to estimate the incidence of paleness or the pale, soft, and exudative condition in the breast muscle of broilers. Customarily, flesh color dominated consumer preference for chickens (18). Supplementation with 0.5 and 1 g/kg of quercetin had no significant effect on the L^*^ value of broilers (19). However, the current study showed that the 0.4 and 0.6 g/kg quercetin group significantly increased the L^*^ value of the thigh muscles (*P* < 0.05). Moreover, alfalfa flavonoids significantly increased the L^*^ value of rabbit meat (20). This probably resulted from the difference in the myoglobin content of the muscle among breeds and difference in concentrations of quercetin (21). Therefore, the result suggested that dietary quercetin supplementation improved the flesh color of the thigh muscles in AA broilers.

Shearing force is an intuitive indicator of muscle tenderness. The lower the shear force, the better the tenderness. Quercetin supplementation significantly increased the shearing force of breast muscle in broilers (19). Our study showed that supplementation with 0.4 g/kg of quercetin significantly reduced the shearing force of the breast and thigh muscles (*P* < 0.05), and supplementation with 0.2 g/kg of quercetin reduced the shearing force of the breast muscle (*P* < 0.05) (Table 5). The difference could have resulted from the difference in breeds, age, or muscle composition of broilers (22).

The drip loss is a quantitative indicator of muscle hydration. The lower the drip loss, the stronger the muscle water-holding capacity. Muscle pH affects the muscle water-holding capacity, which was significantly negatively correlated to drip loss (23, 24). Supercritical CO_2_ extraction of *Schisandra chinensis* (SCESC) reduced lipid peroxidation and thus protected the integrity of the membrane, exerted the normal function, and reduced drip loss (25). Our results showed that supplementation with 0.4 and 0.6 g/kg of quercetin significantly decreased drip loss of the thigh muscle (*P* < 0.05), while supplementation with 0.6 g/kg of quercetin also significantly increased the pH_45min_ of the breast and thigh muscles in broilers (*P* < 0.05), and these results corroborated the previous findings of Schafer (23) and Le Bihan-Duval (24).

SCESC significantly increased the protein content of the breast muscle (26). In this study, the protein content of the breast and thigh muscles of the broilers significantly increased in the 0.6 g/kg quercetin group (*P* < 0.05) and the fat content of the breast muscle significantly reduced (*P* < 0.05). The increased protein content of the breast muscle suggested that quercetin partly improved the nutritional value of the breast muscle. Simultaneously, IMP is an important indicator for measuring the meat flavor and juiciness (27). When the muscle samples were heated in water, fat produced obvious meat flavor. The content of IMP was also closely related to water-holding capacity; the present study showed that supplementation with 0.4 and 0.6 g/kg of quercetin significantly increased the IMP content of the breast and thigh muscles of the broilers (*P* < 0.05), together with drip loss, and it suggested that the decline in drip loss resulted from increasing the IMP content (26).

Color, odor, tenderness, juiciness, and flavor are mostly considered the most important sensory criterion in poultry products. Quercetin enhanced the meat quality of the thigh muscle without adverse effects on color and sensory characteristics in broilers (16). Moreover, quercetin supplementation modified the sensory quality of lamb and goat meat, respectively (27, 28). Among these degradation products, IMP has a positive and even synergistic impact on umami taste (29). Our data showed that the 0.4 g/kg quercetin group showed a significantly increased score of juiciness (*P* < 0.05); the 0.6 g/kg quercetin group showed significantly increased scores of meat color, tenderness, and juiciness (*P* < 0.05); better sensory evaluation was accompanied with increasing quercetin. The results of IMP in this experiment were supported by Khan et al. (29), who found that the content of IMP was directly associated with tenderness and juiciness.

### The mechanism of quercetin ameliorates carcass characteristics and chicken quality in broilers

The previous results of transcriptome sequencing showed that the AMPK signaling pathway is one of the main signaling pathways of lipid metabolism. We previously reported 505 differentially expressed genes of the AMPK signaling pathway we found in the quercetin treatments compared with the control and is the first differentially expressed gene signaling pathway (30).

The AMPK signaling pathway regulates fatty acid synthesis and fatty acid oxidation by controlling lipid metabolism (31, 32) and acts as an energy sensor by regularly responding to cellular energy demands *via* sensing the balance in the AMP-to-ATP ratio (33). Liraglutide is shows to reduce visceral fat mass by inducing beige fat development in obese mice through AMPK signaling (34). In this study, supplementation with 0.4 and 0.6 g/kg of quercetin significantly increased the expression of AMPK_α1_ mRNA in the breast muscle of broilers (*P* < 0.05). The change in muscle pH is one of the most significant changes that occur during abatage and affects meat quality attributes such as texture, color, and water-holding capacity. IMP contributes to the pleasant and fresh flavor of the meat (35). Our results that quercetin affected pH and IMP through increasing the expression of AMPK_α1_ mRNA in the breast muscle of broilers were supported by Shen et al. (36).

These results show that the PI3K/PKB (AKT) signaling pathway participated in adipogenesis (37–39). Taken together, the results are consistent with findings of previous research showing that quercetin improved lipid metabolism and reduced abdominal fat deposition by activating the PI3K/PKB signaling pathway (40). Our results showed that the mRNA expression of PI3K and PKB significantly increased in the 0.4 and 0.6 g/kg quercetin group. Together with the results of AMPK_α1_ in this experiment, quercetin activated the PI3K/PKB (AKT)/AMPK_α1_ signaling pathway, affected fatty acid synthesis and fatty acid oxidation, and thus improved chicken quality.

Fatty acid synthesis and fatty acid oxidation are a complex transcriptional cascade, including the downstream activation of PPARγ, CPT1, HMGR, SREBP1, and L-FABP, which facilitate the activation of the transcription of genes connected to the phenotype of adipocytes (29, 41). Previous studies have shown that blue honeysuckle berry (BHBE) promoted the phosphorylation of AMPK and further reduced the expression of lipogenic transcription-related genes PPARγ and SREBP1, while AMPK inhibitor attenuated the suppressive effect of BHBE on lipogenesis (42).

The transcription factor peroxisome proliferator-activated receptor (PPARγ) plays a key role in regulating adipogenesis and is expressed in the late stages of differentiation. A previous study showed that 50% *Polygonum cuspidatum* ethanol extract (PEE) alleviated lipid accumulation on 3T3-L1 adipocytes and downregulated the mRNA and protein production of adipogenesis-related SREBP1 and PPARγ (43). CPT1 adjusts the β-oxidation of fatty acids by catalyzing the conversion of fatty acyl-CoA into fatty acylcarnitine in mitochondria (44). Instant fermented teas are shown to heighten energy expenditure by increasing CPT1 expression (45). Berteroin was found to significantly increase the expression of mitochondrial fatty acid oxidation-related genes CPT1 and PPARγ, and the phosphorylation of AMPK in HepG2 cells (46). Peroxisome proliferator-activated receptor (HMGR) is a rate-limiting enzyme for cholesterol synthesis. It was found that *Schisandra chinensis* fruit (SF) extract may decrease lipid accumulation by upregulating AMPK and downregulating HMGR (38). A study showed that salsalate reduces atherosclerosis through AMPK, which inhibits fatty acid and cholesterol synthesis through the phosphorylation of HMGCR in mice (47). Sterol regulatory element-binding protein (SREBP1) is found to particularly involve in the activation of the genes controlling fatty acid metabolism and *de novo* lipogenesis. A study showed that berberine may prevent lipid metabolism disorders by decreasing the expressions of SREBP1 and SREBP2 and increasing the expression of AMPK_α1_ (48). The effect of DLBS3733 on lipogenesis and cholesterologenesis is revealed by its activity to increase phosphorylated AMPK and repress the expressions of total SREBP and HMGCR in HepG2 cells (49). Previous studies reported that liver-type fatty acid-binding protein (FABP1/L-FABP) is an important candidate gene for traits of intramuscular and abdominal fat in poultry, and that an FABP1 polymorphism is significantly associated with the deposition of intramuscular and abdominal fat in poultry (50, 51). FABP1 has been found in the cytosol, nucleus, and mitochondria and has multiple roles. Livias reported that due to the role of FABP1 in trafficking fatty acids to the nucleus and also its ability to bind PPARs, the decrease in placentas from women with pre-gestational obesity altered pathways related to placental lipid handling and fatty acid metabolism (52). Our study showed that supplementation with 0.6 g/kg of quercetin group significantly increased the expression of CPT1 and PPARγ (*P* < 0.05) and decreased the expression of HMGR, SREBP1, and FABP1 (*P* < 0.05) by activating AMPK, and thus prevented lipid deposition and promoted lipid transport and lipid β-oxidation.

The correlation analysis showed that there was a significant positive correlation between the pH_45min_ and mRNA expression of PI3K, PKB, AMPK_α1_, SREBP1, and FABP1 (*P* < 0.05); there was also a significant positive correlation between drip loss and mRNA expression of HMGCR (*P* < 0.05); shear force and mRNA expression of PPARγ (*P* < 0.05); and L^*^ value and mRNA expression of CPT1 (*P* < 0.05).

## Conclusion

**Figure 3:**
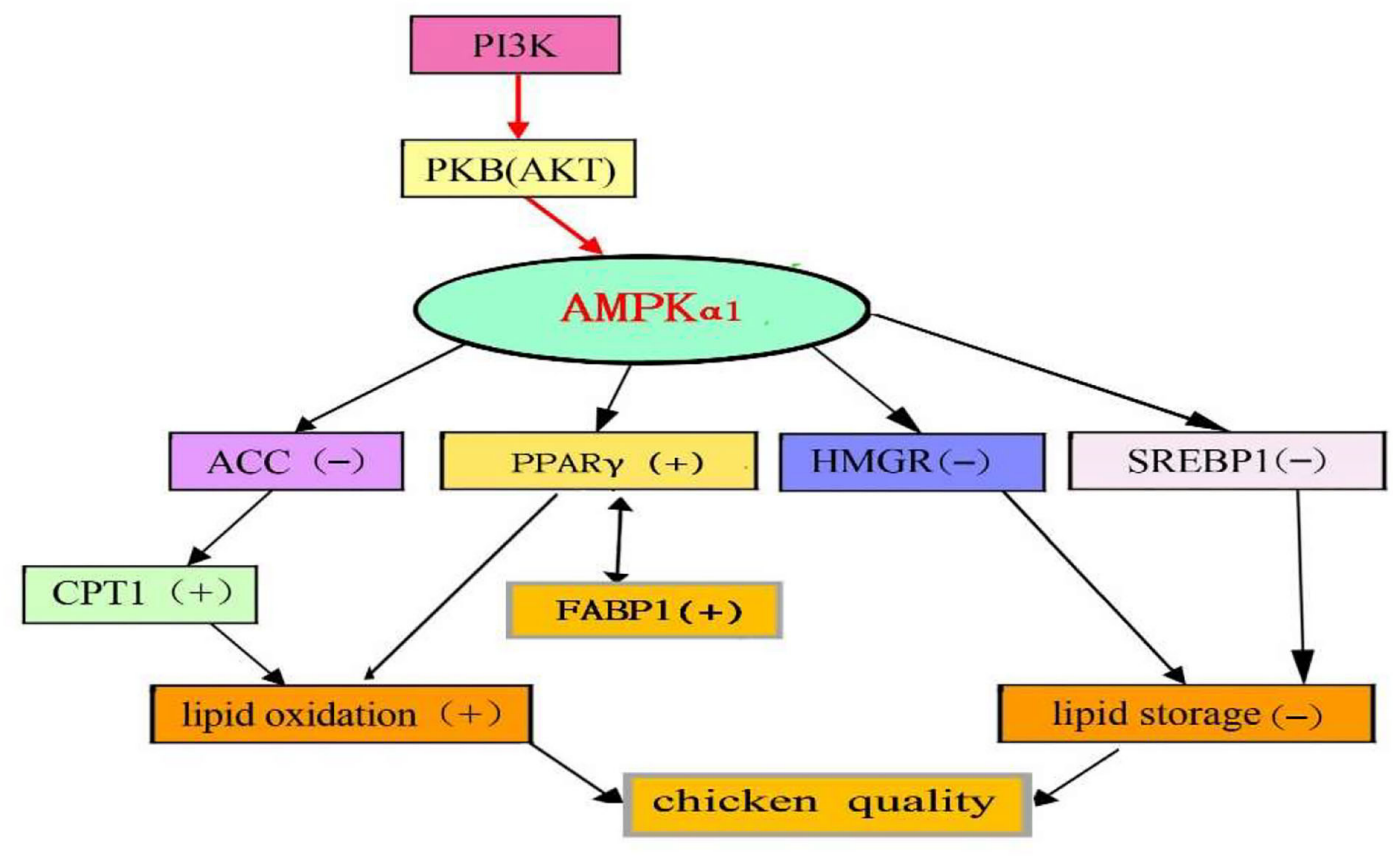
Proposed model of AMPK actions on gene expressions in the breast muscle of chickens supplemented with quercetin [(–), down, (+), up] change.

This study demonstrated that quercetin ameliorates the meat quality and protected against lipid oxidation and deposition by activating the PI3K/PKB/AMPK_α1_ signaling pathway in the breast muscle of broilers (Figure 3). However, as a widespread flavonoid, quercetin is a safe and dietary supplement based on its broad range of biological effects in animals. Quercetin could be used as functional additive ingredients to ameliorate chicken quality. Future studies should investigate the optimal benefits of quercetin, especially in dosing regimens and adjuvants that may amplify any perceived bioactive effects of quercetin *in vivo*.

## Data availability statement

The datasets presented in this study can be found in online repositories. The names of the repository/repositories and accession number(s) can be found in the article/supplementary material.

## Ethics statement

The animal study was reviewed and approved by HEI Animai Management Certificate No. 11928.

## Author contributions

MW and YL participated in the design of the study and critically revised the first manuscript. BW, HW, and SZ provided some technical support for the experiment. JL, MD, and HL performed the experiments and participated in the statistical analysis. YL modified the manuscript and approved the submitted version. All authors contributed to the article and approved the submitted version.
